# Image recognition of traditional Chinese medicine based on deep learning

**DOI:** 10.3389/fbioe.2023.1199803

**Published:** 2023-07-21

**Authors:** Junfeng Miao, Yanan Huang, Zhaoshun Wang, Zeqing Wu, Jianhui Lv

**Affiliations:** ^1^ School of Computer and Communication Engineering, University of Science and Technology Beijing, Beijing, China; ^2^ Business School, Ezhou Vocational University, Ezhou, Hubei, China; ^3^ School of Pharmacy, Xinxiang Medical University, Xinxiang, China; ^4^ Pengcheng Laboratory, Shenzhen, China

**Keywords:** traditional Chinese medicinal, deep learning, classification, image recognition, network models

## Abstract

Chinese herbal medicine is an essential part of traditional Chinese medicine and herbalism, and has important significance in the treatment combined with modern medicine. The correct use of Chinese herbal medicine, including identification and classification, is crucial to the life safety of patients. Recently, deep learning has achieved advanced performance in image classification, and researchers have applied this technology to carry out classification work on traditional Chinese medicine and its products. Therefore, this paper uses the improved ConvNeXt network to extract features and classify traditional Chinese medicine. Its structure is to fuse ConvNeXt with ACMix network to improve the performance of ConvNeXt feature extraction. Through using data processing and data augmentation techniques, the sample size is indirectly expanded, the generalization ability is enhanced, and the feature extraction ability is improved. A traditional Chinese medicine classification model is established, and the good recognition results are achieved. Finally, the effectiveness of traditional Chinese medicine identification is verified through the established classification model, and different depth of network models are compared to improve the efficiency and accuracy of the model.

## 1 Introduction

Chinese medicinal materials are particularly important in the growth of traditional Chinese medicine, which plays an irreplaceable role in treating diseases (Chen and He, 2022). It is also an important component of the most complete traditional medical system in the world today (Xing et al., 2020). Traditional Chinese medicine is also a continuous understanding of medicinal plants that grow in nature. After so many years of development, there are more and more varieties of traditional Chinese medicine, most of which are herbaceous plants, as well as some animal products, minerals, etc (Chen et al., 2021). However, there are also many different types of sub varieties under the same variety, so effective methods are needed to identify the varieties and quality of traditional Chinese medicine. Therefore, the identification and classification of traditional Chinese medicine has always been a hot issue that urgently needs further improvement (Hao et al., 2021). The traditional methods for evaluating the quality of traditional Chinese medicinal materials mainly rely on empirical identification, which can effectively reflect the type and quality of Chinese medicinal materials (Zhou et al., 2021). However, traditional empirical identification is achieved through the subjective feelings and relevant knowledge reserves of professionals (Wang et al., 2020). However, in practical applications, this manual recognition method is cumbersome, inefficient, and the judgment criteria often have subjectivity, posing a risk of misjudgment, affecting the safety and effectiveness of clinical medication, and seriously hindering the healthy development of the traditional Chinese medicine industry (Chu et al., 2022). Therefore, exploring an intelligent, efficient, and high-precision method for identifying traditional Chinese medicine is of great practical significance.

With the rapid development of internet technology and supercomputing, artificial intelligence has gradually spread throughout various fields of human production and life (Wang et al., 2019). In the era of intelligent development, humans are constantly trying to obtain more information from images in better ways. By using computer image recognition technology, a traditional Chinese medicine classification and recognition system is established to efficiently classify and organize a large number of images (Xu et al., 2021). To a certain extent, it can help professionals identify their types, reduce workload, improve work efficiency and recognition accuracy. The deep learning methods in traditional image recognition mainly extract low-level information such as color, texture, shape, etc. It relies on artificially designed features and is difficult to extract high-level semantic features of the image (Zhao et al., 2022). In recent years, deep learning has sparked a wave of enthusiasm in the academic community, widely applied in image recognition and achieved breakthrough results (Wang et al., 2023). It can effectively map low-level features to high-level fields, obtain more essential feature representations, and ensure higher recognition rates while providing more convenient operations. Deep learning is a reliable method to improve the efficiency and accuracy of identifying traditional Chinese medicine types (Han et al., 2022). Therefore, this article uses convolutional neural networks to complete the identification and classification of traditional Chinese medicine.

The main work and structure of this article are as follows.

(1) This article studies the development of traditional Chinese medicine recognition, identifies the targets that need to be recognized, and constructs a dataset of traditional Chinese medicine images.
(2) This article preprocesses images of traditional Chinese medicine. By normalizing, graying, and image denoising operations, the quality of traditional Chinese medicine images has been improved. Then, in order to solve the problem of a small number of image datasets for traditional Chinese medicine, data augmentation techniques are used to ensure the adequacy of the image dataset.
(3) This article uses an improved ConvNeXt network for feature extraction and classification of traditional Chinese medicine. This method adds a Stacked ACMix module to the second ConvNeXt Block layer inside the ConvNeXt network to ensure the adequacy of low-dimensional feature extraction in the image. Then, a Stacked ACMix module is added to the last ConvNeXt Block layer inside the ConvNeXt network to ensure high-dimensional feature extraction and achieve sufficient information fusion. In the linear layer of the Head, the Stacked FFN network is added to ensure the specificity of its application in traditional Chinese medicine recognition. Finally, a comparative experiment is conducted on network models of different depths, and a network model with better classification performance is selected.

The remaining part of this article consists of four parts, and Section 2 is the related literature of this article. Section 3 provides a detailed introduction to Convolutional neural network, dataset for medicinal image, and recognition model. Section 4 analyzes the proposed algorithm and its effectiveness through experiments. Finally, the conclusion of this article is summarized.

## 2 Literature review

The combination of computer technology and traditional Chinese medicine identification and recognition has begun and in traditional Chinese medicine detection has received some research. Wu et al. (Wu et al., 2016) obtained the odor and color characteristics of traditional Chinese medicine using electronic nose and computer vision, and then used BP and SVM methods for classification and recognition; Hussein et al. (Hussein et al., 2020) identified forest plants using discriminant analysis, random forests, and support vector machines, showing the feasibility of using extracted traits to identify plants. The method described above has achieved good results to some extent, but has the following limitations: shallow features are the feature information that directly comes from image pixels without high-level semantics, and are easily affected by the detection environment. In practical applications, recognition reliability is poor (Sun and Qian, 2016). At the same time, feature extraction algorithms are established for visual shapes, colors, and textures, and then fused. The algorithm is complex and fails to consider the correlation between shapes, colors, and textures, resulting in poor results and reduced recognition efficiency. Therefore, the detection technology of traditional Chinese medicine based on image processing and pattern recognition is greatly limited in practice, and a new identification method is urgently needed.

Recently, great advances have been achieved in artificial intelligence, image recognition, and other technologies, greatly promoting related technologies. In order to develop a fast, automatic, and accurate image recognition system, convolutional neural network (CNN) has received widespread attention (Artzai et al., 2019). In particular, CNN can complete the extraction of low-level features to high-level semantics (Kattenborn et al., 2019), significantly increasing the accuracy of identification and identification in various fields. Park et al. (Park et al., 2017) proposed a player evaluation model based on deep learning to analyze the impact on baseball leagues. Batchuluun et al. (Batchuluun et al., 2018) used CNN models to recognize human bodies in motion. Hansen et al. (Hansena et al., 2018) implemented the recognition of individual pigs using deep learning methods, with an accuracy rate of 96.7%. Fricker et al. (Fricker et al., 2019) proposed a hyperspectral tree image recognition method using CNN models. Altuntas et al. (Altuntaş et al., 2019) used CNN models to automatically identify haploid and diploid maize seeds. Zhang et al. (Zhang et al., 2019) proposed a network model based on void convolution for identifying six common cucumber plant diseases. While related research on traditional Chinese medicine recognition and deep learning methods have also begun to be combined, Lee et al. (Lee et al., 2015) studied a deep learning method that used deconvolution networks (DN) to learn recognition features from 44 different plant images, proving that learning features through CNN was superior to traditional manual features. Hu et al. (Hu et al., 2018) suggested and obtained excellent classification results with a multi-scale fusion convolutional neural network for plant leaf identification. Zhu et al. (Zhu et al., 2018) improved the deep convolutional neural network by dividing the initial picture into smaller pictures and loading them into the network, achieving rapid and accurate classification of plant leaves. Park et al. (Park et al., 2021) designed a multi rate three-dimensional convolutional neural network. This method enhanced the image and classified it through a joint fusion classifier. The experimental results had high accuracy. Liu et al. (Liu et al., 2022a) designed a network for recognizing animal fur. This network was based on feature fusion and fully utilized the texture information and inverted feature information of fur images, improving accuracy on the dataset. Jeong et al. (Jeong et al., 2020) proposed a facial expression recognition method. This method used three-dimensional convolution to simultaneously extract spatial and temporal features. Then, these features were combined through a joint fusion classifier to complement each other. Kim et al. (Kim et al., 2019) proposed a new method for FER systems based on hierarchical deep learning. This method integrated features extracted from appearance networks with geometric features in a hierarchical structure and improves accuracy on the dataset.

## 3 Image recognition of traditional Chinese medicine based on deep learning

**FIGURE 1:**
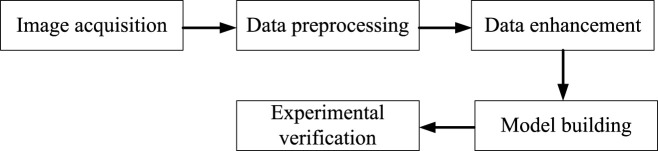
Maximum pooling and average pooling

Deep learning technology has been gradually applied to Natural language processing, voice, image and other aspects (Wang et al., 2021), and has made breakthrough progress in the application field of artificial intelligence, as well as excellent achievements in many fields (Wang et al., 2022). In order to solve the image recognition of traditional Chinese medicine, deep learning technology is used to complete the recognition of traditional Chinese medicine, which not only effectively solves the problems of misjudgment and low efficiency caused by human factors, but also has high detection and recognition rate, convenient operation, and greatly saves time and labor costs. The main process is shown in Figure 1.

### 3.1 Convolutional neural network

Convolutional neural network can learn the features of images and are widely used in computer vision tasks such as image classification and object detection (Namatēvs, 2017). In recent years, with the continuous deepening of research, the structure of convolutional neural networks has been continuously improved, resulting in many classic network structures (Hubel and Wiesel, 1962; Fukushima and Miyake, 1982). Convolutional neural network is mainly composed of convolution layers, pooling layer, and fully connectivity layer. By repeatedly stacking convolution and pooling layers, convolutional neural network can extract local and global feature information in images, and then complete tasks such as classification or regression through fully connectivity layer.The convolutional neural network (LeCun et al., 1998) has five hierarchical structures.

(1) Input layer

The input layer of convolutional neural network is used to input raw or preprocessed data into the network.

(2) Convolution layer

Convolution layer is a linear computational layer that uses a series of convolutional kernels and multi-channel input data for convolution. Convolution is a fundamental operation in analytical mathematics, in which all the functions used to perform operations on input data are called convolutional kernels. Convolutional operations refer to the process of the convolutional kernel making small weighted sums at various positions of input data in the form of sliding windows. In practical operations, the convolution layer will use different numbers of convolution cores for convolution operations, and the weight of each convolution core will always remain unchanged. This procedure could decrease the number of parameters, speed up neural network convergence, and shorten training time.

(3) Pooling layer
  The pooling layer further processes feature maps and effectively reduces and filters the size and valuable feature information within them. Convolution layer and full connectivity layer are difficult to converge and compute feature maps that control size during the operation process, resulting in the appearance and extraction of some useless features. So during the process of changing image size, the pooling layer can effectively reduce the redundancy of features by filtering and processing useless features.

**FIGURE 2:**
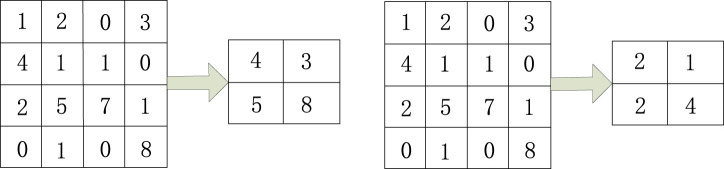
Maximum pooling and average pooling.

Pooling methods generally include maximum pooling and average pooling. The maximum pooling selection preserves the maximum value in the local area of the feature map as the result value after pooling. For mean pooling, the local average value of the feature map is selected as the pooling result. Figure 2 shows the pooling operation

(4) Full connectivity layer

The full connectivity layer is used in the network structure as a way to connect with various nodes in the previous layer using full connectivity. Its function is to transform the image feature maps output in the convolutional layer into vector form. At the same time, the dimension of the image feature map is transformed into a one-dimensional vector and mapped into the sample label space, which is then input into the classifier in the network model for recognition and classification (Szegedy et al., 2015). The parameters of the fully connectivity layer account for a significant proportion of all parameters in the convolutional neural network model, resulting in a large number of parameters that consume a significant amount of storage and computational resources. And it is easy to cause the problem of overfitting of network model, which has an important impact on the improvement of network training efficiency.

(5) Output layer

The output layer is used to output numerical values calculated by convolutional neural network. The form of output varies for different problems.

### 3.2 Construction of medicinal material image dataset

**FIGURE 3:**
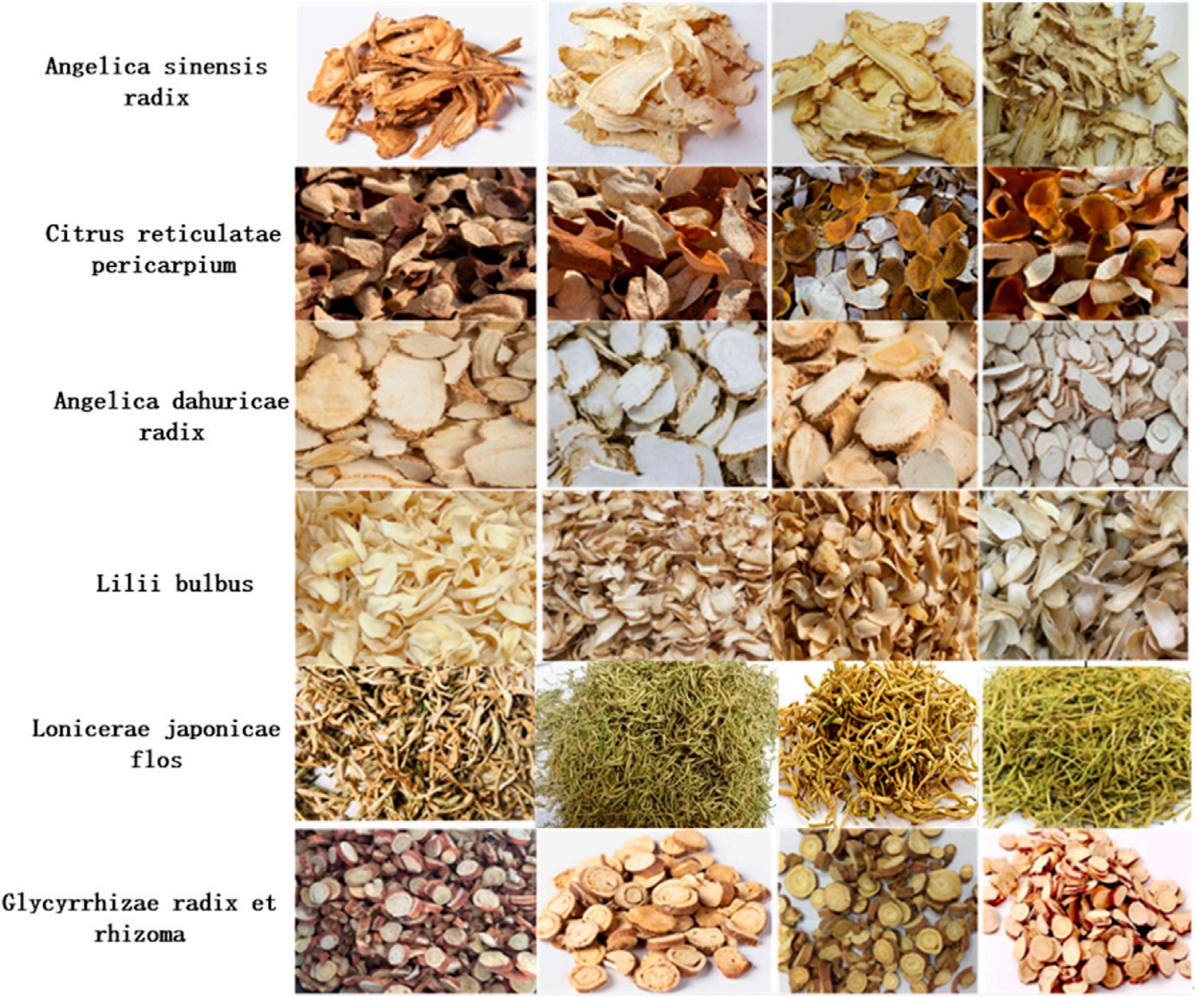
Image samples.

Due to the lack of publicly available datasets for traditional Chinese medicine, and the relatively high requirement for dataset size in deep learning classification tasks, only sufficient datasets can fully train and improve the model, obtaining excellent models with strong generalization ability (Krizhevsky et al., 2012). Therefore, the dataset selected in this article is shown in Figure 3, which mainly consists of six categories: angelica sinensis radix, citrus reticulatae pericarpium, angelica dahuricae radix, lilii bulbus, lonicerae japonicae flos, glycyrrhizae radix et rhizome. These data mainly come from filming, network crawling, and so on.

### 3.3 Image data preprocessing

Image samples are the data foundation for image recognition algorithm research. Compared to directly inputting the original image into the network for training, the preprocessed data samples are easier to train and have better training results (He et al., 2016a). In addition, a comprehensive database can help improve the generalization ability and robustness of the network. In deep learning, image quality affects recognition accuracy, and the number of images affects network generalization performance.

#### 3.3.1 Image data normalization

In deep learning, the image is usually normalized before model training, and normalizing the image adjusts the size of the feature values to a similar range. If not normalized, the gradient value will also be larger when the feature value is larger, and smaller when the feature value is smaller (Simonyan and Zisserman, 2014). Therefore, in order to make the model training converge smoothly, the image is normalized and the feature values of different dimensions are adjusted to similar ranges[36]. In this paper, the linear function conversion method is selected for image normalization, because the linear function conversion method does not involve the calculation of distance measurement and covariance, which is relatively simple and suitable for image normalization in image processing. The formula is as follows.

$$b=\left(a-\mathrm{Max}\right)/\left(\mathrm{Max}-\mathrm{Min}\right)$$ (1)

In the formula, b is the output of image pixel values, a is the input of image pixel values, and max and min are the maximum and minimum pixel values.

#### 3.3.2 Grayscale image data

Image grayscale is a grayscale technique that processes the three channels of RGB with color images into a single channel grayscale image (Shotton et al., 2013). In the process of color image processing, it is necessary to sequentially process the three RGB colors, which not only increases the time cost but also increases the processing pressure on hardware devices. Therefore, in order to solve the problems caused by color image processing, it is necessary to perform grayscale preprocessing operations on color images. Single channel grayscale images also have the effect of reflecting the contrast between the chromaticity and brightness levels of the entire image. Each type of color image has its own morphological feature representation, and each color image adopts different grayscale processing methods. The grayscale processing methods for color images include component grayscale, maximum grayscale, average grayscale, and weighted average grayscale (Huang et al., 2021). Here, the weighted average method is applyied to grayize the medicinal image.

#### 3.3.3 Image data denoising

During the processing of images, it is inevitable that they will be affected by various intensity signals, which will affect the quality of the image and disrupt the correlation between the content structure and pixels in the image, which is not conducive to further analysis of the image (Han et al., 2022). The goal of image denoising is to improve the quality of the specified image and solve the problem of image quality degradation caused by noise interference. This article selects median filtering to denoise Chinese herbal medicine images. Median filtering is a nonlinear image processing technique that preserves more details of the image and does not cause image blurring issues. And it does not replace the central target pixel with the average of the pixels in the template, but sorts all the pixels in the template, taking the median of the sorted template pixel sequence as the value of the target pixel. In addition, median filtering not only has a significant effect on eliminating isolated noise points, but also has a good removal effect on slightly dense noise points. And it also has a particularly good removal effect on salt and pepper noise. Overall, the denoising effect of median filtering is superior to other algorithms (Hao et al., 2022).

### 3.4 Data enhancement

The problem of image recognition based on deep learning usually requires large-scale training data samples, otherwise the problem of overfitting will occur because the model is too complex and the amount of data is too small. In fact, it is difficult for people to directly collect the amount of data that meets the requirements of deep neural network training. The collection and screening of training data is a very time-consuming and mental task, and the workload is extremely large. Therefore, this article adopts the method of image data enhancement to increase the number of image samples (Wang et al., 2023). First, image rotation and mirror symmetry are performed on the traditional Chinese medicine image. Image rotation refers to rotating all pixels of an image at an angle of 0-360 around the center of the image. Mirror symmetry refers to the exchange of all pixels in the image by using the vertical line in the image as the axis. Then, the image difference method is used to change the size of the original image to different sizes, and new images are randomly cropped on the scaled image (Wang et al., 2021).

### 3.5 Construction of traditional Chinese medicine image recognition model

Convolutional neural network has received widespread attention in the field of computer vision in international academia and industry in recent years due to their relatively fast development compared to other image processing methods, In the current development of cutting-edge technology and the resolution of research hotspots, convolutional neural network has become the most effective and practical tool for solving practical engineering problems in the field of computer vision. Over time, many mainstream convolutional neural network models have emerged, such as GoogLeNet (Szegedy et al., 2015), VGG (Simonyan and Zisserman, 2015), ResNet (He et al., 2016b) and ConvNeXt (Liu et al., 2022b). This article mainly builds the model based on ConvNeXt.

#### 3.5.1 Building an identification model

**FIGURE 4:**
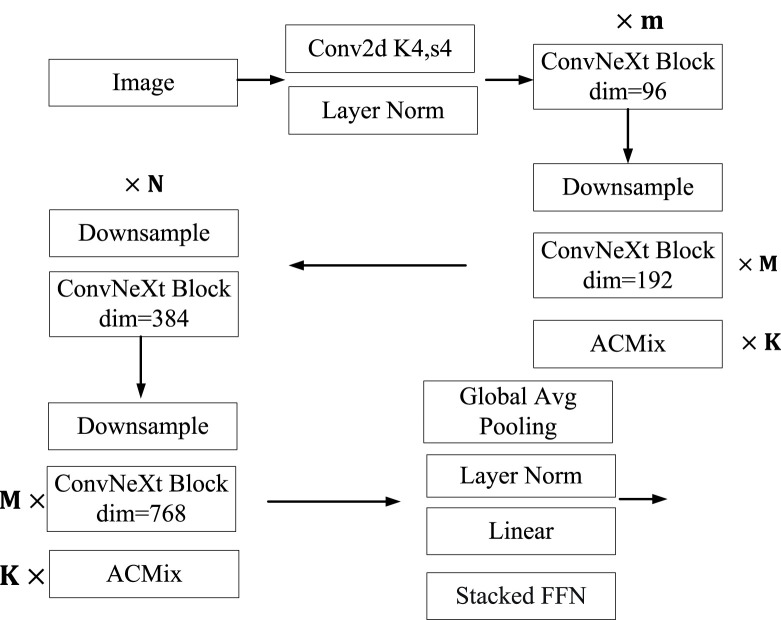
Recognition model.

The convolutional neural network algorithm has been the mainstream idea in the field of image recognition to solve the problem of image recognition, until the emergence of the Vision Transformer (Dosovitskiy et al., 2020) algorithm, which transferred the Transformer idea from the NLP field to the image field. Afterwards, a series of new algorithms such as ViT, Swin Transformer, and DeiT are born. With the use of Transformer algorithm in images, there is a growing trend in research to replace convolutional neural networks with Transformer algorithm. Until the birth of the ConvNeXt algorithm, it is once again demonstrated that convolutional neural networks are still an effective algorithm in the field of image recognition. This article is mainly based on the ConvNeXt algorithm and improves it. It mainly adds ACMix to the ConvNeXt algorithm, which includes convolution operations and Multi-Head Self Attention operations. It can extract the image features of traditional Chinese medicine through two different operations, and finally fuse the features to output the fused results. At the same time, it adds a stacked FFN structure to the Head layer of ConvNeXt to output the category of traditional Chinese medicine. The specific algorithm framework is shown in Figure 4.

The traditional Chinese medicine recognition model constructed in this article has the following structure. The image size of the traditional Chinese medicine after image preprocessing is 224×224×3 . It first passes through a two-dimensional convolutional layer with Kernel 4 and Strike 4, and then performs Layer Norm normalization on the convolutional images. Then the image will be compressed into a feature image of 56×56×96 . The image passes through m ConNeXt Block layers again, with a Dimension of 96. The feature image output from the ConNeXt Block layer is downsampled, and the width and height of the downsampled image are reduced by half, resulting in a final feature image size of 28×28×96 . Through the stacked ConvNeXt Block layer with a Dimension of 192, the output features have become 28×28×192 . The image features output by the Stacked ConvNeXt Block layer are input into the Stacked ACMix module, which allows for varying degrees of extraction of features from the image through both Attention and Convolution operations. By integrating the advantages of two different frameworks, the extracted features can focus on both local features and global features through the Attention layer. The feature size output by ACMix is still 28×28×192 . Later, the Downsampling of this feature becomes 14×14×192 . The feature image of 14×14×192 is fed into the Stacked ConvNeXt Block layer with a Dimension of 384, and the output feature is 14×14×384 . The feature map of 14×14×384 is downsampled again to become 7×7×384 . The image features of 7×7×384 are processed through the Stacked ConvNeXt Block layer and output as a feature image of 7×7×768 . The feature image is input into the Stacked ACMix. The output is 7×7×768 . The output features use global average pooling again, and then pass through the Layer Norm layer. After normalization through the Layer Norm layer, it is inputted into the Stacked FFN network, and finally the classification head is output to complete the image recognition of traditional Chinese medicine.

#### 3.5.2 Parameter setting

In the network designed in this article, the setting of relevant parameters has a significant impact on the accuracy of classification. The EPOCH hyperparameter in this paper is 100. The parameters of M,N and K are parameters that need to be optimized during operation. The different values of M, N and K directly determine the number of layers in the network. This article uses a pre-trained model. ConvNeXt has 5 models including ConvNeXt-nano model, ConvNeXt-Tiny model, ConvNeXt-Small model, ConvNeXt-Base model, and ConvNeXt-Large model. This article is an improvement on the ConvNeXt Tiny model. The values of K selected in this article are a set composed of 1,2 and 3. The initial value of learning rate γ is 0.0002. The maximum width of the network is 768.

## 4 Experiment and result analysis

### 4.1 Experimental environment and design

The experimental environment for this article is Linux operating system, Intel Core CPU, 16 GB of running memory, and the GPU is NVIDIA RTX3060TI. The deep learning framework pytorch is used to recognize medicinal herbs. This article conducts experimental design on the proposed model. Firstly, based on the actual situation of traditional Chinese medicine recognition, the accuracy of classification is selected to measure whether the experimental results meet the requirements. Accuracy refers to the percentage of correctly predicted quantities in the entire dataset. The analysis of the experimental results is the recognition accuracy of the proposed model on the dataset.

This article is an improvement on the ConvNeXt network architecture by adding Stacked ACMix to improve network performance. At the same time, it improves the ability to extract information from features. Due to the inclusion of CNN and Attention modules in ACMix, it is possible to extract local information from images as well as global information features through the Multi Head Self Attention module. Secondly, a comparative experiment is conducted on network models of different depths, with models of 20-layer blocks, 22-layer blocks, and 24-layer blocks designed for comparison, and a network model with better classification performance is selected.

### 4.2 Analysis of experimental results

**FIGURE 5:**
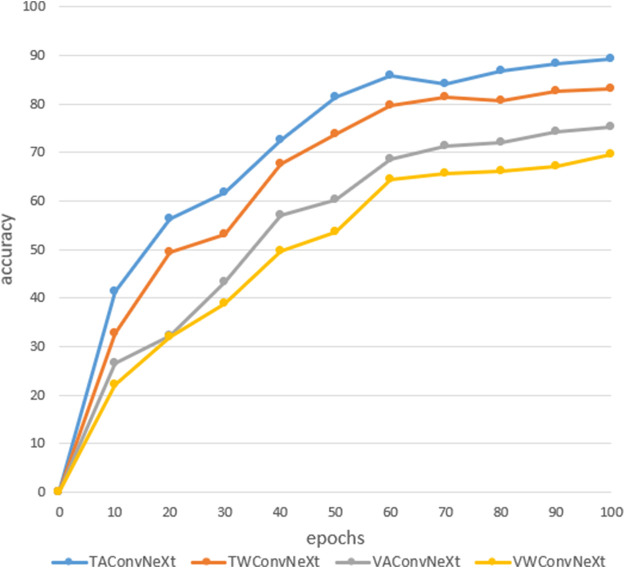
Recognition results.

In order to improve the recognition rate of the model, the Stacked ACMix module is added when building the network model. In order to verify the effect of the Stacked ACMix module, a 20-layer block neural network model is used, and the GELU is the activation function. A comparative experiment is designed to determine whether to add the Stacked ACMix module. Our data set has 7,853 images, including 5,497 images in the training set, 1,571 images in the verification set, and 785 images in the test set. Figure 5 shows the corresponding recognition rates trained after adding the Stacked ACMix module in the network model, verified after adding the Stacked ACMix module, trained without adding the Stacked ACMix module, and verified without adding the Stacked ACMix module layer.

From the analysis of the above experimental results, it can be concluded that adding the Stacked ACMix module to the network model significantly improves the recognition rate. After adding the Stacked ACMix module, the training and verification rates reached 89.3% and 83.1%, respectively, while the training and verification rates without the Stacked ACMix module were 75.3% and 69.6%. Therefore, the Stacked ACMix module will improve the performance of the model and improve its recognition accuracy.

**FIGURE 6:**
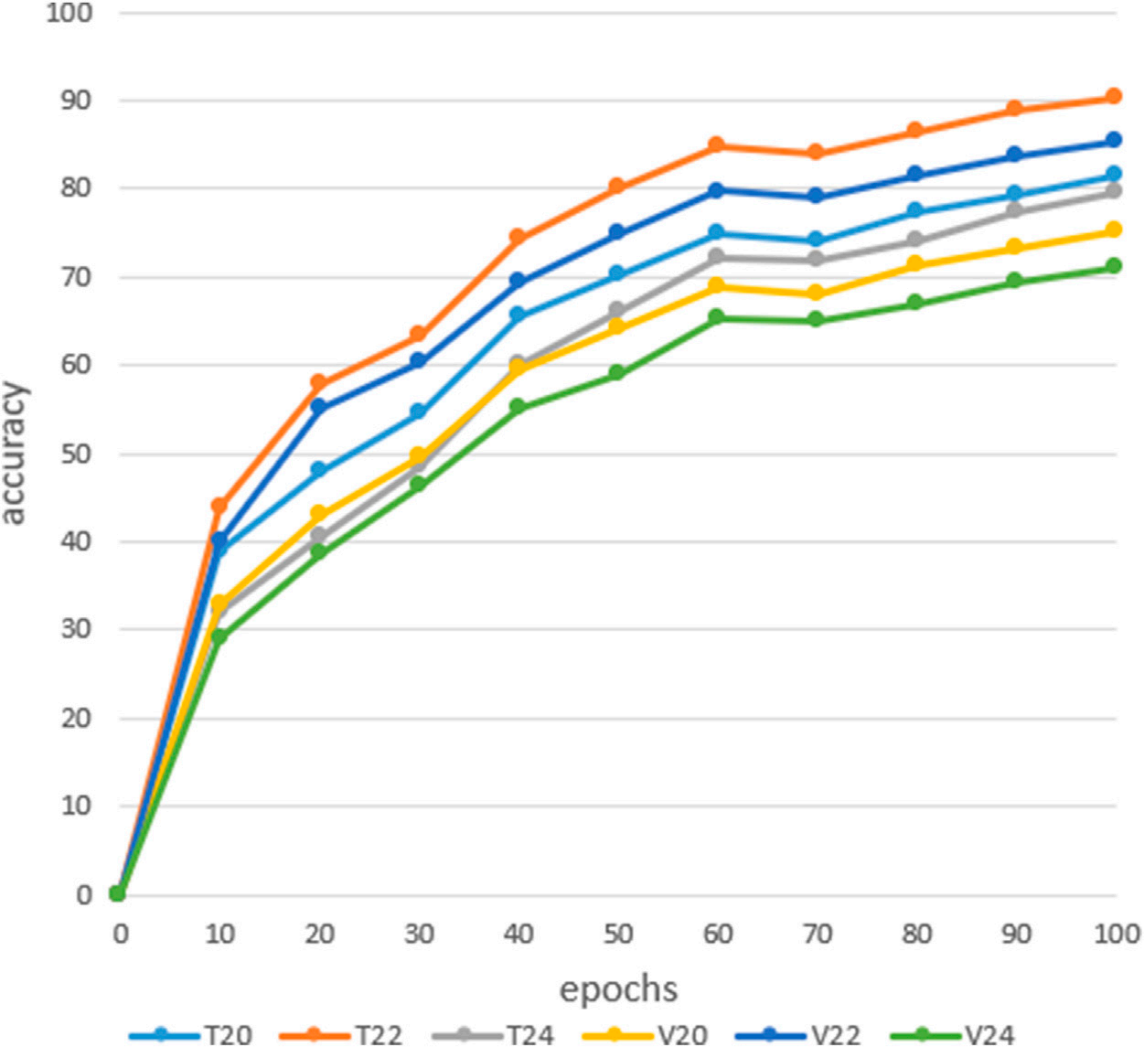
Training results.

In CNN, insufficient depth of the model will make it impossible to correctly fit the features of the data. If the model is too deep, it will require learning a large number of weight parameters to increase the training time of the model (He et al., 2016). Complex models are prone to overfitting in the training process, reducing the generalization ability of the model, so it is necessary to combine the actual situation to construct the depth of the model.In order to explore the recognition rate between different depth models, this article conducted medicinal image recognition experiments on three different models: 20-layer, 22-layer, and 24-layer. The results in Figure 6 shows the training and verify rates of the 20-layer, 22-layer, and 24-layer networks. From the analysis of the following experimental results, it can be concluded that when the depth of the network model is 22-layers, the recognition rate of the model is optimal, with a training rate of 91.3% and a verify rate of 85.2%, respectively. Secondly, when the depth of the model is 20 layers, the training rate and verify rate are 81.6% and 75.3%, respectively. Finally, when the depth of the model is 24 layers, the classification performance of the model is poor, with training and verify rates of 79.6% and 71.1%, respectively. Therefore, when the depth of the model is 22-layers, the model has good classification performance. Supplementary Table S1 shows the result of a model based on a 22-layer convolutional neural network on a test set is 80.5%. From the above analysis, it can be concluded that when the model depth is 22-layers, the model has a higher recognition rate on the training set, validation set, and test set. Therefore, we use a model depth of 22-layers as the number of layers for the convolutional neural network model.

## 5 Conclusion

With the development of the times, traditional Chinese medicine identification methods have been continuously inherited and innovated, and deep learning technology has been applied to the identification process of traditional Chinese medicine to obtain more objective, simple and convenient practical identification methods. This article first introduces the categories of traditional Chinese medicine, and then preprocesses the medicinal image through normalization, grayscale, noise reduction, and other methods to improve the effectiveness of the medicinal image recognition algorithm. Data enhancement technology is used to increase the data size of the medicinal image and solve the problem of a small image dataset. Finally, this article uses the improved ConvNeXt to extract features and classify and differentiate traditional Chinese medicine. By adding ACMix and stacked FFN networks to the ConvNeXt network, its feature extraction ability is improved. The efficiency and accuracy of the model are improved by setting different depth network models for comparison. The specific recognition effect of traditional Chinese medicine images is tested using the constructed neural network model.

## Data Availability

The raw data supporting the conclusion of this article will be made available by the authors, without undue reservation.
