# Supplementary material for: Image recognition of traditional Chinese medicine based on deep learning
Source: Front Bioeng Biotechnol. 2023 Jul 21;11:1199803. doi: 10.3389/fbioe.2023.1199803 (PMC10402920; doi:10.3389/fbioe.2023.1199803)
Supplement: Supplementary file 1 [file Table1.docx]

| Epoch | 10 | 20 | 30 | 40 | 50 | 60 | 70 | 80 | 90 | 100 |
| --- | --- | --- | --- | --- | --- | --- | --- | --- | --- | --- |
| Test accuracy | 36.4% | 51.3% | 55.6% | 65.6% | 72.5% | 74.6% | 74.1% | 76.4% | 78.6% | 80.5% |

Table1. Test accuracy
